# Innovative Real‐Time Flow Sensor Using Detergent‐Free Complex Emulsions with Dual‐Emissive Semi‐Perfluoroalkyl Substituted *Α*‐Cyanostilbene

**DOI:** 10.1002/advs.202304108

**Published:** 2023-09-13

**Authors:** Narani Rakesh, Hsiung‐Lin Tu, Po‐Chun Chang, Sofani Tafesse Gebreyesus, Che‐Jen Lin

**Affiliations:** ^1^ Department of Chemistry National Dong Hwa University Shoufeng 974301 Taiwan; ^2^ Institute of Chemistry Academia Sinica Nangang Taipei 115201 Taiwan

**Keywords:** aggregation‐induced emission enhancement, complex emulsions, excimer emission, microfluidics, sensors

## Abstract

In this study, the potential of complex emulsions is investigated as transducers in sensing applications. Complex emulsions are stabilized without external detergents by developing a novel *α*‐cyanostilbene substituted with PEG and semi‐perfluoroalkyl chain (**CNFCPEG**). **CNFCPEG** exhibits unique variable emission properties depending on its aggregation state, allowing dual blue and green emissions in complex emulsions with hydrocarbon‐in‐fluorocarbon‐in‐water (H/F/W) morphology. The green excimer emissions result from the self‐assembly of **CNFCPEG** at the fluorocarbon/water interface, while the blue emission observed is due to aggregation in the organic phase. A novel flow‐injection method is developed by incorporating complex emulsions with **CNFCPEG** into multiple‐well flow chips (MWFC). Iodine is successfully detected in a mobile aqueous solution by monitoring morphology changes. The findings demonstrate that self‐stabilized complex emulsions with MWFC hold great promise for real‐time sensing without costly instruments.

## Graphical abstract

A semi‐perfluoroalkyl substituted amphiphilic *α*‐cyanostilbene, **CNFCPEG**, exhibits intriguing aggregation‐dependent dual fluorescence properties, blue and green emission at low and high aggregation levels, respectively. Complex emulsions with **CNFCPEG** are stable without external surfactants and show morphology‐dependent emission at the fluorocarbon/water interface. A multiple‐well flow chip (MWFC) is developed, incorporating droplets to monitor the iodine content in the mobile solution.

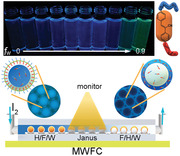

## 1 Introduction

Luminescent materials are essential in emitting different colors in response to environmental changes such as polarity, temperature, pH, or chemical substances.^[^ ^1^ ^]^ *α*‐cyanostilbenes are of great interest as a multifunctional building unit in organic optoelectronic and photonic devices, bioimaging, photoswitches, and sensors.^[^ ^1^, ^2^ ^]^ *α*‐cyanostilbenes are aggregation‐induced enhanced emission (AIEE) fluorophores that are nearly non‐emissive in dilute solution whereas become highly emissive in aggregates or solid states. The solid states containing *α*‐cyanostilbene exist stimuli‐responsive fluorescence in which fluorescence color can be transformed upon mechanical grinding, thermal annealing, or vapor fuming.^[^ ^3^ ^]^

Numerous luminogens only exhibit single‐mode AIEE, including monomer or excimer emission. Excimer emission requires proper alignment of molecular packing and sufficient *π* orbital overlap of the dye molecules, whereas monomer emission needs the central luminophore to maintain a certain distance. However, controlling the molecular orientation and distance in solution is a significant challenge since molecules exhibit higher degree of “freedom” in solution than their solid‐state counterpart. Tang's work has showcased the utilization of interfacial self‐assembly as a viable method for achieving single‐mode AIEE.^[^ ^4^ ^]^ However, precise manipulation of the interface to elicit distinct emission remains unexplored. To address the aforementioned issue, complex emulsions with multiphase liquid are potential platforms for accurately controlling the molecular assembly of AIEE molecules in solution.

Complex emulsions composed of hydrocarbon (H) and fluorocarbon (F) oils^[^ ^5^ ^]^ dispersed in water (W) exhibit dynamic internal morphologies,^[^ ^6^ ^]^ which show distinct optical^[^ ^7^ ^]^ and luminescent properties.^[^ ^8^ ^]^ Typically, these complex emulsions have emerged as a promising sensing platform^[^ ^9^ ^]^ by altering surfactant composition or effectiveness.^[^ ^10^ ^]^ Fluorescence as the signal readout can differentiate H/F/W and F/H/W with high contrast regardless of droplet orientation. Swager and coworkers reported morphology‐dependent complex emulsions based on green‐fluorescence protein chromophores sensitive to environmental hydrogen bonding.^[^ ^8^ ^]^ Likewise, Zeininger and coworkers reported polymeric surfactants, including tetraphenylethylene dye, and demonstrated enhancing emission of Janus droplets.^[^ ^8^ ^]^ Controlling the interfacial area between oil and water is crucial to manipulating emissions. We envision the interfacial area as an ideal platform for controlling self‐assembly in solution and incorporating sensing elements.

To this goal, we designed an amphiphilic α‐cyanostilbene, **CNFCPEG**, substituted with a semi‐perfluoroalkyl chain and tetraethylene glycol and used the F/W interface of complex emulsions as a platform to demonstrate self‐assembly. We propose that the amphiphilic *α‐*cyanostilbene with semi‐perfluoroalkyl chain can strongly assemble on the F/W interface to stabilize complex emulsions and exhibit dynamic self‐assembly as morphology varies. The droplets exhibit blue emission in the hydrocarbon phase and green emission at the F/W interface. The green emissions disappear when the morphology changes from H/F/W to F/H/W. Using an inherent surfactant instead of adding surfactant in a continuous phase enables the use of complex emulsions in fluid systems.

Note that fluid systems may randomize and perturb droplets, thus interfering with observation and quantitative analysis. To address this challenge, microfluidics are essential in sensing applications to enable rapid and real‐time measurement of molecular interactions.^[^ ^11^ ^]^ For example, Savagatrup et al. successfully demonstrated a sensing platform combining poly(dimethylsiloxane) (PDMS) microfluidics and complex droplets.^[^ ^12^ ^]^ Herein, we develop a multiple‐well flow chip (MWFC) containing wells with a similar diameter as droplets’. This device allows us to real‐time monitor droplets at a higher flow rate than a single‐well chip. Combination of complex emulsions with **CNFCPEG** and MWFC, we quantitatively detect iodine in a fluid as a proof of concept.

Iodine is a micronutrient essential for thyroid function and human health,^[^ ^13^ ^]^ but excess or deficient iodine can have adverse effects. Therefore, monitoring and maintaining iodine levels in the environment is crucial for human health.^[^ ^14^ ^]^ Gold standard methods require high capital expense, such as inductively coupling liquid chromatography mass spectrometry (ICP‐MS) and gas chromatography mass spectrometry (GC‐MS).^[^ ^14^, ^15^ ^]^ Sample pretreatment restricts these methods to real‐time monitoring in natural water bodies and municipal wastewater. An emerging strategy for iodine sensing is the flow‐injection method via detecting absorbance or luminescence.^[^ ^16^ ^]^ However, developing an efficient and convenient method for continuously monitoring I_2_ remains challenging. This study presents a novel approach to rapidly and quantitatively sense iodine using self‐stabilized complex emulsions in MWFC. This sensory platform is low‐cost and offers easy optical read‐out without the need for expensive instruments.

## 2 Results and Discussion

### 2.1 Synthesis and Photophysical Properties

**Scheme 1:**
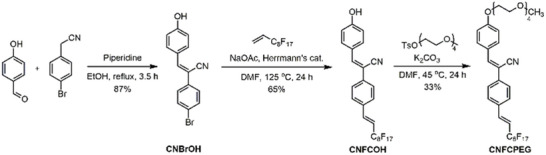
Synthesis scheme of **CNFCPEG**.

**Scheme** **1** shows the synthesis of amphiphilic *α*‐cyanostilbene (Supporting information). Briefly, 4‐hydroxybenzaldehyde and 4‐bromophenylacetontrile first underwent the Knoevenagel condensation to generate **CNBrOH**. Subsequently, a Heck reaction‐based protocol reported by Swager^[^ ^17^ ^]^ was adopted to synthesize **CNFCOH**. **CNFCOH** then reacted with tetraethylene glycol mono methyl ether tosylate to produce **CNFCPEG** via an S_N_2 reaction.

**Figure 1:**
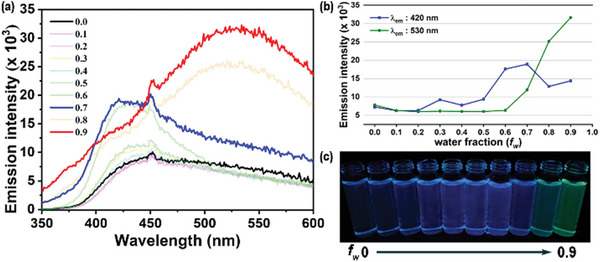
a) The emission spectra of **CNFCPEG** in THF/water mixed solution (5 × 10^−5^ m). b) The intensity of 430 and 530 nm at water fraction (*f*_w_) from 0−0.9. c) The photo of CNFCPEG in THF/water mixed solution under 365 nm irradiation.

**Figure** **1** shows the emission spectra of **CNFCPEG** in the THF/water mixed solutions (5.0×10^−5^ m) with water fraction (*f*_w_) from 0 to 0.9. The solution shows weak blue emission at low water fraction, and the intensity grows twice at *f*_w_ = 0.7 due to aggregation‐induced emission enhancement. Surprisingly, the emission color turns green at *f*_w_ = 0.8 and 0.9. We tentatively attribute the green emission to the excimer emission in the highly aggregated state. Indeed, Park and coworkers have reported that the solids packing of *α*‐cyanostilbene might lead to the emission changes from blue to green, with the interconnected nature being monomer and excimer, respectively.^[^ ^3c^ ^]^ The emission color changes while increasing the aggregation extent have been observed in the platinum complexes.^[^ ^18^ ^]^ Nonetheless, such a phenomenon is uncommon for *α*‐cyanostilbene in solutions. In our study, the fluorous interactions are essential to direct the molecular alignment to form excimer emission.^[^ ^19^ ^]^ The emission of **CNBrOH** and **CNFCOH** aggregates is blue and green, respectively (Figure S1, Supporting Information). The variation of aggregation emission color of **CNFCPEG** is a synergistic effect of the hydrophilic PEG and fluorophilic fluoroalkyl interactions. We thus investigated the relationship between emission intensity and **CNFCPEG** concentration at *f*_w_ = 0.8 (Figure S2, Supporting Information). The emission of excimer exhibits a gradual increase at low concentrations, followed by a rapid growth between 11–19 µm, and then a slow increase at higher concentrations. This observation is similar to the profile of micelle formation^[^ ^20^ ^]^ inin that **CNFCPEG** may form micelles and ordered molecular alignment while aggregating.^[^ ^21^ ^]^

**Figure 2:**
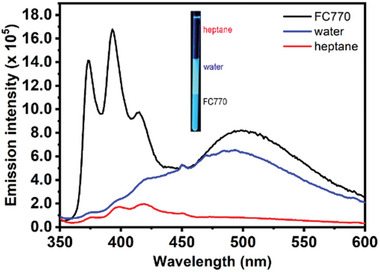
The emission spectra of **CNFCPEG** (2.5×10^−5^ m) in heptane and FC770 mixed solutions in the presence of water. The inlet is the photos of **CNFCPEG** in heptane/FC770/water mixed solutions.

We dissolved **CNFCPEG** in heptane (5 × 10^−5^ m) and mixed it with equal amount of FC‐770 in an NMR tube. **CNFCPEG** primarily partitions in heptane, as shown in Figure S3 (Supporting Information), and it emits a stronger blue emission in heptane compared to FC‐770 (Figure S4, Supporting Information). Adding water leads to the formation of emulsions in the middle aqueous layer. Surprisingly, **CNFCPEG** shows green excimer emission in FC770 and water, while the heptane remains blue (**Figure** **2**). Based on the aforementioned experiments, it is reasoned that **CNFCPEG** self‐assembles in water and forms emulsions due to its amphiphilic nature. The rising emission intensity of FC770 evidences an increased partition of **CNFCPEG** that may form aggregate and/or micelle upon adding water. The excimer emissions in the FC770 are likely due to the tendency of the fluoroalkyl chain to form *J*‐aggregates in fluorous solvents.^[^ ^19c^ ^]^

### 2.2 Complex Emulsions Containing CNFCPEG with Tween‐20 and FS‐30

**Figure 3:**
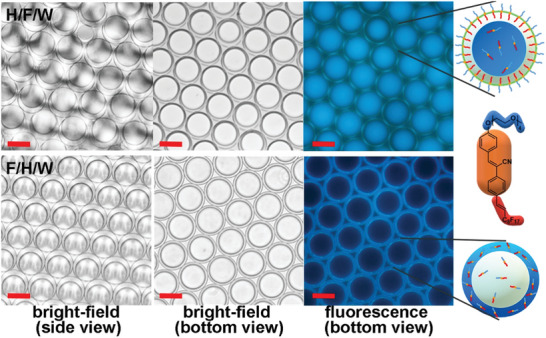
Microscopic bright‐filed side view, bright‐field, and fluorescence bottom view of H/F/W emulsions in 0.5 wt.% FS‐30 and F/H/W emulsions in 0.5 wt.% Tween‐20 (scale bar: 50 µm). The schematic illustration depicts **CNFCPEG** partition alongside the emission colors exhibited by the double emulsions.

In this study, we aimed to prepare mono‐dispersed double emulsions using a RayDrop emulsion device. We emulsified preheated heptane and FC‐770 with **CNFCPEG** (2.5 × 10^−5^ m) into aqueous solutions containing 0.5 wt.% Tween‐20. The resulting double emulsions exhibited an average diameter of 65 µm in the aqueous solutions with external detergents of 0.5 wt.% FS‐30 and 0.5 wt.% Tween‐20, respectively (**Figure** **3**). We observed that in the H/F/W morphology, the inner heptane phase emitted blue light, while the FC770/water interface showed green emission. This green emission was due to the formation of *J*‐aggregates of **CNFCPEG**s, which aligned parallelly with their PEG chains oriented toward the water phase and the fluorocarbon chains directed toward the fluorocarbon layer.^[^ ^22^ ^]^ In contrast, the F/H/W emulsions only displayed blue emissions from the outer heptane layer without the excimer emissions at the H/W interface. Notably, the emissions detected in the bulk solution within an NMR tube primarily originated from water and FC770, whereas the emissions observed from droplets were predominantly attributed to the heptane layer. This distinction can be attributed to the variance in density, resulting in the extraction of fluorous and water layers of **CNFCPEG** from heptane within the NMR tube. We schematically summarize the partition of **CNFCPEG** in complex emulsions and emission colors in Figure 3.

**Figure 4:**
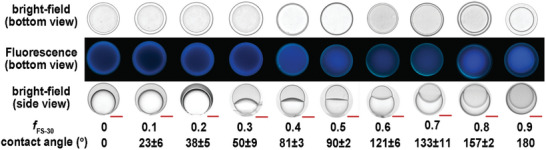
The bright bottom view, fluorescence bottom view, and bright side view of complex emulsions with different contact angles. ($f_{\mathrm{FS}-30}=\frac{\left[\mathrm{FS}-30\right]}{\left[\mathrm{FS}-30]+[\mathrm{Tween}-20\right]}\;$) (scale bar: 20 µm).

We then investigated the emission properties by varying morphology, as shown in **Figure** **4** and Figure S5 (Supporting Information). The F/H/W emulsions gradually change to Janus and H/F/W emulsions upon increasing the fraction of FS‐30 (*f*_FS‐30_). The green emission ring appears when the morphology converts from F/H/W to Janus. The complex emulsions exhibited dynamic partition of **CNFCPEG** that more F/W interfacial area facilitates **CNFCPEG** to participate from the heptane to F/W interface. It is worth mentioning that the incorporation of **CNFCPEG** results in the Janus morphology as a snowman shape rather than a spherical shape without **CNFCPEG**. The snowman shape indicates reduced F/H surface area and increased interfacial tension. This phenomenon becomes particularly prominent probably due to the utilization of a concentrated **CNFCPEG** solution in emulsions, leading to the formation of aggregates or assemblies, particularly at the oil/water interface. The heterogeneous solutions can consequently result in elevated surface tension at the F/H interface. Similar snowman Janus droplets have been observed in the case of complex emulsions with polymer surfactants^[^ ^23^ ^]^ and liquid crystals.^[^ ^24^ ^]^

### 2.3 Complex Emulsions Containing CNFCPEG without External Detergents

**Figure 5:**
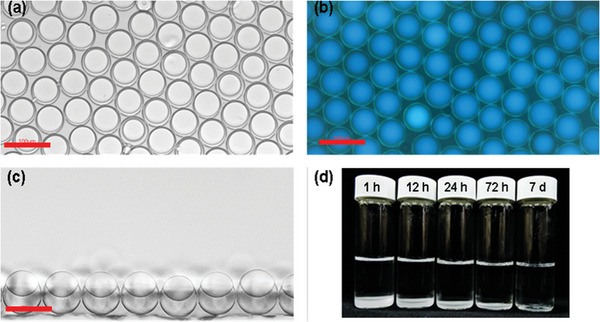
Microscopic a) bright bottom view, b) fluorescence bottom (scale bar 100 µm), and c) bright side view images (scale bar 50 µm) of the monodispersed droplets. d) The number of complex emulsions after 1 h, 12 h, 24 h, 72 h, and 7 d.

The amphiphilic **CNFCPEG** as internal surfactant allows us to prepare complex emulsions without adding external surfactants or detergents in a continuous phase. As shown in **Figure** **5**, the monodispersed droplets containing 5 × 10^−5^ m **CNFCPEG** are H/F/W double emulsions featuring contact angles of 138 ± 4^o^ without Tween‐20 and FS‐30. It suggests that the PEG and fluoroalkyl chains stabilize the F/W more than the H/W interface. Consequently, we measured the surface tension using a pendant drop tensiometry. The results also show that the FC‐770 with **CNFCPEG** exhibited lower surface tension in contact with air than heptane with **CNFCPEG**. (Figure S6, Supporting Information) The green emission ring at the F/W interface further supports the assembly of **CNFCPEG**. We then investigated the physical stability of the emulsions. The mono‐dispersed droplets remained stable without contacting other droplets for 4 h (Figure S7, Supporting Information). Yet over time, these droplets started to coalesce, and the number of droplets decreased, eventually becoming a clear solution after 7 days (Figure 5d). Despite lacking longer‐term stability, current emulsion stability already allows us to develop a sensing system with continuous flow based on freshly prepared complex emulsions.

### 2.4 Morphology and Emission Color Changes of Complex Emulsions Containing CNFCPEG with Iodine

**Figure 6:**
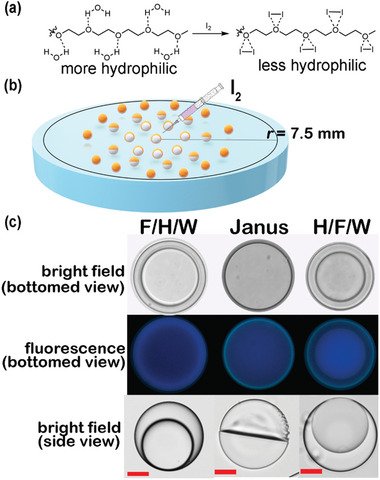
a) The reaction scheme of PEG upon adding iodine to reduce hydrophilicity. b) The schematic drawing of the morphology changes dependent on the iodine diffusion. c) The bright bottom view, fluorescence bottom view, and bright side view of complex emulsions in the petri dish (scale bar: 20 µm).

**Figure** **6**a illustrates the PEG group's hydrophilicity reduction during its reaction with iodine.^[^ ^25^ ^]^ This characteristic serves as the basis for iodine content detection. The experimental setup involved placing emulsions in DI water (3 mL) within a petri dish. Upon deposition, the droplets spread to form circular patterns with a 1.5 cm diameter (Figure 6b). Introducing iodine at a concentration of 10 ppm into the circle's center caused an instantaneous morphology shift from H/F/W to F/H/W. This morphological transformation toward Janus was observed within a certain radius from the injection center, while the edge of the circle retained the H/F/W configuration. The progressive morphological shift signifies that the reaction between iodine and PEG is faster than the diffusion process. Notably, the emission colors associated with each morphology shown in Figure 6c closely resemble those in Figure 4. It is crucial to highlight that the emulsion morphology transition transpired without coalescence or rupture, distinguishing it from typical morphological shifts. The reduction in hydrophilicity at the F/W interface is attributed to the effects of the PEGs‐iodine reaction.^[^ ^25^, ^26^ ^]^ To counteract the elevated surface tension, the **CNFCPEG** within the heptane phase dynamically participates at the H/W interface, consequently stabilizing the droplets. This dynamic partitioning could elucidate the observed morphological changes, mitigating droplet rupture. The increasing H/W interface results from the stabilizing influence exerted by **CNFCPEG** from the heptane. Furthermore, we observe that the surface of the H/W interface on the Janus droplets displays an irregular texture in Figure 6c, potentially serving as evidence for interactions between PEG and iodine. The observed spherical shape can be attributed to the de‐assembly of aggregates, which contributes to the heightened F/H interfacial tension.

**Figure 7:**
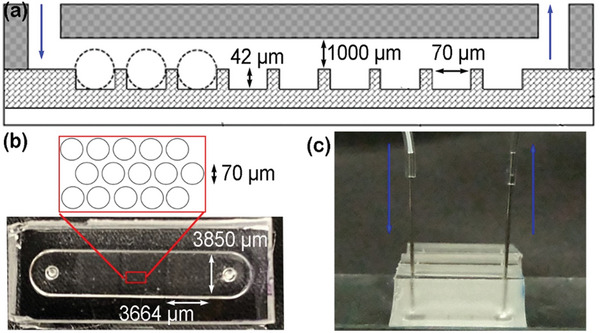
a) The schematic drawing from the side of MWFC. The photo of the MWFC from the b) top and c) side.

Encouraged by the promising iodine sensing with the complex emulsions, we thus developed a PDMS multiple‐well flow chip (MWFC) (**Figure** **7**a–c). The chip features an array of microwells (diameter = 70 µm; height = 42 µm) and a 1000 µm height channel. Video S1 (Supporting Information) demonstrates the droplet loading process in which each well contains a single droplet. The monodispersed droplets (55–65 µm) stay effectively in these microwells even at an 8 mL min^−1^ flow rate. The droplets remain H/F/W morphology at a 0.5 mL min^−1^ flow rate using DI water over 3 min. (Video S2, Supporting Information)

**Figure 8:**
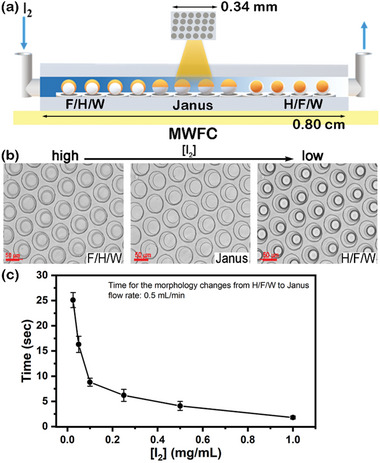
a) The schematic drawing of the method to monitor morphology in MWFC. b) The morphology changes of complex emulsions in the chip under the flow of iodine_(aq)_ (0.1 mg mL^−1^) at the flow rate of 0.5 mL min^−1^. (scale bar: 50 µm) c) The relation between time to morphology change and iodine concentration at the flow rate of 0.5 mL min^−1^.

To demonstrate the concept of real‐time sensing, we injected iodine_(aq)_ and monitored the morphological change of these complex emulsion droplets within a specific region (0.34 × 0.34 mm) at the center of MWFC as shown in **Figure** **8**a. Remarkably, while adding iodine solution, the droplets remain stable and change their morphology to Janus and F/H/W (Figure 8b; Video S3, Supporting Information). We quantified the characteristic time required for the droplets in the center of MWFC to change from H/F/W to Janus morphology and found a significant decrease in this characteristic time as the concentration of iodine increased (Figure 8c). A slower flow rate corresponded to longer characteristic times, as listed in Table S1 (Supporting Information). Overall, the characteristic time and the observed spatial evolution within MWFC may offer valuable quantitative insights into analytes.

Existing droplet‐based sensors focus on single batches of samples due to the reliance on external detergents. Furthermore, the accurate interpretation of signal read‐outs, such as changes in scattering pattern,^[^ ^9^, ^27^ ^]^ transparency,^[^ ^28^ ^]^ and emission intensity,^[^ ^9^, ^24^, ^29^ ^]^ demands the use of well‐aligned and stable droplets. This requirement is crucial for reducing the noise of the measurements. Our research introduces an advancement by emphasizing the significance of self‐stabilized droplets with internal surfactants. These droplets empower us to employ a flow‐injection method without external detergents. Notably, this reported strategy is based on monitoring changes in droplet morphology as the primary signal read‐out, which can be conveniently achieved using a readily accessible, basic microscope. Instead of using costly instruments like mass spectrometry, our methodology holds promise for application even in resource‐constrained settings. We highlight the potential of using the MWFC with self‐stabilized complex emulsions for future studies aimed at real‐time monitoring.

### 2.5 Potential Limitations of the Current Method

We have successfully demonstrated the real‐time detection of iodine utilizing complex emulsions within MWFC. However, it is essential to acknowledge and address potential limitations inherent in our current methodology.

We have employed **CNFCPEG** as an internal surfactant to stabilize the emulsions. The presence of a higher concentration of **CNFCPEG** results in enhanced emulsion stability. Moreover, **CNFCPEG** acts as a receptor for iodine, implying that its abundance influences the interaction with iodine. Notably, excessive utilization of **CNFCPEG** may also lead to a morphological shift needing excess iodine, which subsequently impacts the lower detection limit. On the other hand, the MWFCs in our setup comprise PDMS, a material known for its ease of fabrication and cost‐effectiveness. Nevertheless, using PDMS introduces a challenge as it interferes with luminescence transmittance, thus hindering the acquisition of satisfactory emission images and intensity from the MWFC.

Introducing a receptor other than **CNFCPEG** is crucial to surmounting these limitations. This strategy would significantly enhance the detection sensitivity and selectivity for analytes of interest. We also recommend implementing an MWFC design with a thinner PDMS layer or employing other chip fabrication materials such as glass. Such adjustments can potentially ameliorate luminescence transmittance, thereby improving emission image quality and intensity, leading to a more sensitive detection scheme.

## 3 Conclusion

In conclusion, we synthesized an amphiphilic *α*‐cyanostilbene, **CNFCPEG**, which contains a semi‐perfluoroalkyl group. **CNFCPEG** shows blue emission at low aggregation levels and turns to green excimer emission at high aggregation levels. Our findings indicate that incorporating **CNFCPEG** into complex emulsions results in morphology‐dependent green emission at the F/W interface. More importantly, **CNFCPEG** also functions as an internal surfactant that stabilizes complex emulsions without needing external surfactants. To demonstrate the capabilities of our platform, we developed a sensing system that uses an MWFC filled with **CNFCPEG** complex emulsions. As a proof‐of‐concept, we successfully monitored the iodine content within the mobile aqueous solution in real‐time based on the morphology change. This technology can potentially benefit the field of chemical and biological sensing by enabling real‐time monitoring of analytes in fluid environments.

## 4 Experimental Section

### Polydispersed Complex Emulsions Preparation

Hydrocarbon oil (heptane) and fluorocarbon oil (FC‐770) were heated above the upper critical temperature to form a homogeneous solution. The mixture was transferred by a pipette into an aqueous solution with hydrocarbon surfactant Tween 20 (0.5 wt.%) or fluorocarbon surfactant Capstone FS30 (0.5 wt.%). The solution was vigorously vortexed for 5 s to generate polydispersed droplets in the 50–150 um diameter range.

### Monodispersed Complex Emulsions Preparation

Hydrocarbon oil (heptane) and fluorocarbon oil (FC‐770) in a 15‐mL Falcon tube and the continuous aqueous phase with Tween 20 (0.5 wt.%) in a 50 mL Falcon tube was heated in an aluminum heating block (50 °C). The microfluidic system includes Falcon tubes, the microfluidic flow controllers (Flow EZ, Fluigent), and the RayDrop Single Emulsion Device (Fluigent). Controlling the pressure generates monodisperse droplets of 50–80 µm. The **CNFCPEG** (4 × 10^−7^ mmol) was dissolved in 500 µL heptane and 500 µL FC770 as the dispersed phase.

### Pendant Drop Measurement

**CNFCPEG** was individually dissolved in heptane and FC‐770 at a concentration of 5×10^−5^ m. Interfacial tension measurements were taken using a Kruss Drop Shape Analyzer DSA100S. The surface tension was obtained using the pendant drop tensiometry technique with Young–Laplace fitting method.

### Fabrication of Multi‐Well Flow Chips

The multi‐well flow chip (MWFC) consists of two polydimethylsiloxane (PDMS) layers, namely a top flow layer (L1) and a bottom well layer (L2), bound to a glass substrate. Both L1 and L2 layers were designed using the AutoCAD software (Autodesk, USA). L1 contains a rectangular channel with semicircular ends. The dimensions of the rectangular channel were 16 mm x 3.8 mm x 1 mm (length × width × height), and semicircular ends had a diameter of 1.9 mm and height of 1 mm. L2 contains an array of microwells arranged hexagonally with a diameter of 70 µm and a spacing of 15 µm apart. For the L1 layer, an aluminum mold was fabricated using the high‐precision machining protocol (Institute of Physics, Academia Sinica Taiwan). Meanwhile, a binary photomask mold was used for the L2 layer (M&R Nano Technology Co. Ltd., Taiwan). A standard photolithography protocol was followed to fabricate the L2 master mold. Briefly, the process began by thoroughly washing a 4‐inch silicon wafer. After drying at 105 °C for 5 min, the photoresist (SU‐8 3025, MichroChem, USA) was spun over the wafer at 2000 r.p.m to achieve a height of 42 µm. The wafer then underwent pre‐exposure baking (65 °C for 2 min and 95 °C for 10 min), followed by exposure to mercury‐match light (250 mJ cm^−2^; 10 s) using the dedicated L2 photomask and a mask aligner EV‐620. The processed wafer was then post‐exposure baked (65 °C for 2 min and 95 °C for 5 min), developed, and further baked at 160 °C for 3 h before ready for use.

To fabricate MWFC, the L2 master mold was first pre‐treated with chlorotrimethylsilane to ensure its easy separation from PDMS in the subsequent step. Next, a mixture of 10:1 (base: curing agent) PDMS was prepared and degassed (Thinky ARE‐310 Mixer), followed by spun over the pretreated wafer mold at 500 rpm (Laurell WS‐650 HZ‐23NPP/UD2 Spin coater) and baked in an 80 °C oven for 45 min. To prepare the L1 layer, 20 g of PDMS was prepared and poured over the L1 aluminum mold, followed by degassing for 1 h and baking at 80 °C for 45 min. The L1 PDMS layer was then peeled‐off from the mold, and trimmed, and inlet and outlet holes were punched using a puncher (Syneoco, USA). Meanwhile, the L2 PDMS layer was peeled‐off from the mold, trimmed, and carefully flipped over a clean glass slide. Both layers were then treated by oxygen plasma at the highest RF level for 1 min (Harrick plasma cleaner PDC‐001‐HP), aligned (custom stereo‐Nikon‐SMZ18 microscope with independent *x*‐, *y*‐, and *z*‐alignment controller), bound, and baked at 80 °C for 12 h. The two‐layer PDMS slab was finally bound to a plasma‐treated 25 × 75 mm glass slide and baked for at least 24 h before use.

### Iodine Detection Using MWFC

The MWFC device was first connected to two PTFE microfluidic tubing inlets and outlets. Before injecting droplets, the MWFC was flushed with DI water for 2 min at a 5 mL min^−1^ flow rate to remove air bubbles. Complex emulsions with **CNFCPEG** in an aqueous solution were injected into MWFC. After filling the wells with droplets, MWFC was purged with DI water at 1 mL min^−1^ to remove untrapped droplets in the channel. The MWFC used for iodine sensing was filled with droplets in >98% of wells. A syringe pump injected aqueous iodine solution into MWFC to control the flow rate. A Nexcope NIB410‐FL inverted fluorescence microscope equipped with 10X, 20X, and 40X plan phase objectives and 20X and 40X semi‐Apo FL objectives were used. Droplets were specifically targeted in the center of the MWFC as depicted in Figure S6 (Supporting Information). The time when the morphology changed was recorded and repeated five times.

## Conflict of Interest

The authors declare no conflict of interest.

## Supporting information

Supporting Information

Supplemental Video 1

Supplemental Video 2

Supplemental Video 3

## Data Availability

The data that support the findings of this study are available in the supplementary material of this article.

## References

[1] a) H. Yuan , K. Wang , K. Yang , B. Liu , B. Zou , J. Phys. Chem. Lett. 2014, 5, 2968; 26278244 10.1021/jz501371k

[2] a) P. Mahalingavelar , S. Kanvah , Phys. Chem. Chem. Phys. 2022, 24, 23049; 36128991 10.1039/d2cp02686d

[3] a) S. Shin , S. H. Gihm , C. R. Park , S. Kim , S. Y. Park , Chem. Mater. 2013, 25, 3288;

[4] a) J. Li , Y. Li , C. Y. K. Chan , R. T. K. Kwok , H. Li , P. Zrazhevskiy , X. Gao , J. Z. Sun , A. Qin , B. Z. Tang , Angew. Chem., Int. Ed. 2014, 53, 13518; 10.1002/anie.201408757 PMC4370284 25363745

[5] M. Pavlovic , H. K. Ramiya Ramesh Babu , S. Djalali , Z. Pavlovic , M. Vranes , L. Zeininger , Langmuir 2023, 39, 2152. 36744990 10.1021/acs.langmuir.2c02346

[6] B. D. Frank , S. Djalali , A. W. Baryzewska , P. Giusto , P. H. Seeberger , L. Zeininger , Nat. Commun. 2022, 13, 2562. 35538083 10.1038/s41467-022-30229-3 PMC9091213

[7] a) J. Wang , U. Sultan , E. S. A. Goerlitzer , C. F. Mbah , M. Engel , N. Vogel , Adv. Funct. Mater. 2019, 30, 1907730;

[8] a) C. J. Lin , L. Zeininger , S. Savagatrup , T. M. Swager , J. Am. Chem. Soc. 2019, 141, 3802; 30785273 10.1021/jacs.8b13215 PMC7255052

[9] a) L. Zeininger , E. Weyandt , S. Savagatrup , K. S. Harvey , Q. Zhang , Y. Zhao , T. M. Swager , Lab Chip 2019, 19, 1327; 30896702 10.1039/c9lc00070d PMC6482465

[10] Y. Takahashi , K. Fukuyasu , T. Horiuchi , Y. Kondo , P. Stroeve , Langmuir 2014, 30, 41. 24354334 10.1021/la4034782

[11] a) A. Sridhar , A. Kapoor , P. S. Kumar , M. Ponnuchamy , B. Sivasamy , D.‐V. N. Vo , Environ. Chem. Lett. 2022, 20, 901; 34803553 10.1007/s10311-021-01342-4 PMC8590809

[12] B. Barua , T. J. Durkin , I. M. Beeley , A. Gadh , S. Savagatrup , Soft Matter 2023, 19, 1930. 36807488 10.1039/d3sm00074e

[13] G.‐H. Zhang , L. Zhang , Q.‐H. Zhu , H. Chen , W.‐L. Yuan , J. Fu , S.‐L. Wang , L. He , G.‐H. Tao , ACS Mater. Lett. 2021, 4, 136.

[14] a) A. Saiz‐Lopez , J. M. Plane , A. R. Baker , L. J. Carpenter , R. von Glasow , J. C. Martin , G. McFiggans , R. W. Saunders , Chem. Rev. 2012, 112, 1773; 22032347 10.1021/cr200029u

[15] C. P. Shelor , P. K. Dasgupta , Anal. Chim. Acta 2011, 702, 16. 21819856 10.1016/j.aca.2011.05.039

[16] a) V. V. Kuznetsov , Y. V. Ermolenko , L. Seffar , J. Anal. Chem. 2007, 62, 479;

[17] a) K. Yoshinaga , T. M. Swager , Synlett 2018, 29, 2509;

[18] C.‐J. Lin , Y.‐H. Liu , S.‐M. Peng , T. Shinmyozu , J.‐S. Yang , Inorg. Chem. 2017, 56, 4978. 28406626 10.1021/acs.inorgchem.7b00009

[19] a) E. M. Sletten , T. M. Swager , J. Am. Chem. Soc. 2014, 136, 13574; 25229987 10.1021/ja507848f PMC4577963

[20] a) J. K. Salem , I. M. El‐Nahhal , S. F. Salama , Chem. Phys. Lett. 2019, 730, 445;

[21] G. Ren , L. Wang , Q. Chen , Z. Xu , J. Xu , D. Sun , Langmuir 2017, 33, 3040. 28282144 10.1021/acs.langmuir.6b04546

[22] a) Y. Takahashi , N. Koizumi , Y. Kondo , Langmuir 2016, 32, 683; 26731043 10.1021/acs.langmuir.5b03912

[23] Y. He , S. Savagatrup , L. D. Zarzar , T. M. Swager , ACS Appl. Mater. Interfaces 2017, 9, 7804. 28198607 10.1021/acsami.6b15791

[24] a) A. Concellón , C. A. Zentner , T. M. Swager , J. Am. Chem. Soc. 2019, 141, 18246; 31675218 10.1021/jacs.9b09216

[25] G. Wang , B. Tang , Y. Liu , Q. Gao , Z. Wang , X. Zhang , Chem. Sci. 2016, 7, 1151. 29910871 10.1039/c5sc03907j PMC5975747

[26] S. Hemalatha , B. Chandani , D. Balasubramanian , Spectrosc. Lett. 1979, 12, 535.

[27] a) J. Li , S. Savagatrup , Z. Nelson , K. Yoshinaga , T. M. Swager , Proc. Natl. Acad. Sci. USA 2020, 117, 11923; 32414933 10.1073/pnas.2002623117 PMC7275673

[28] a) S. Nagelberg , L. D. Zarzar , N. Nicolas , K. Subramanian , J. A. Kalow , V. Sresht , D. Blankschtein , G. Barbastathis , M. Kreysing , T. M. Swager , M. Kolle , Nat. Commun. 2017, 8, 14673; 28266505 10.1038/ncomms14673 PMC5344304

[29] L. Zeininger , S. Nagelberg , K. S. Harvey , S. Savagatrup , M. B. Herbert , K. Yoshinaga , J. A. Capobianco , M. Kolle , T. M. Swager , ACS Cent. Sci. 2019, 5, 789. 31139715 10.1021/acscentsci.9b00059 PMC6535765

