# Supplementary material for: Innovative Real‐Time Flow Sensor Using Detergent‐Free Complex Emulsions with Dual‐Emissive Semi‐Perfluoroalkyl Substituted Α‐Cyanostilbene
Source: Adv Sci (Weinh). 2023 Sep 13;10(31):2304108. doi: 10.1002/advs.202304108 (PMC10625100; doi:10.1002/advs.202304108)
Supplement: Supplementary file 1 — Supporting Information [file ADVS-10-2304108-s003.pdf]

ADVANCED SCIENCE
Open Access

Supporting Information

for *Adv. Sci.*, DOI 10.1002/advs.202304108

Innovative Real-Time Flow Sensor Using Detergent-Free Complex Emulsions with Dual-Emissive Semi-Perfluoroalkyl Substituted *A*-Cyanostilbene

*Narani Rakesh, Hsiung-Lin Tu, Po-Chun Chang, Sofani Tafesse Gebreyesus and Che-Jen Lin**

Supporting Information

Innovative Real-Time Flow Sensor Using Detergent-free Complex Emulsions with Dual-Emissive Semi-perfluoroalkyl Substituted α-Cyanostilbene

*Narani Rakesh,[†] Hsiung-Lin Tu,[†] Po-Chun Chang, Sofani Tafesse Gebreyesus, Che-Jen Lin**

Experimental section

General Methods

Heptane HPLC Grade was purchased from J.T. Baker™, FC770 Fluorinert from 3M Tween-20 from Sigma Aldrich, Capstone FS-30™ from Apollo Scientific Ltd, and iodine from Sigma-Aldrich. Deionized (DI) water was used directly from a Milli-Q system. NMR spectra were recorded using a Bruker Advance 400 MHz NMR spectrometer and were referenced to the proton resonances resulting from incomplete deuteration of NMR solvent. Spire mixer model no-5100 from Pantech Instruments. Absorption spectra were carried out by Agilent Cary 60 UV/Vis spectrophotometer. Emission studies were measured by Edinburgh FLS 920 spectrometer equipped with a 450 W Xenon lamp as the excitation source. Emulsions were placed in a 0.2-mm short-path-length cell from Shufu Instruments to collect droplet's emission spectra. Bright-field and fluorescence images were taken with a Nexcope NIB410-FL inverted fluorescence microscope equipped with plan phase objectives 10X, 20X, 40X; semi-Apo FL objectives 20X, 40X. The side-view images were taken with a SOPTOP BH200 biological microscope lying on the bench.

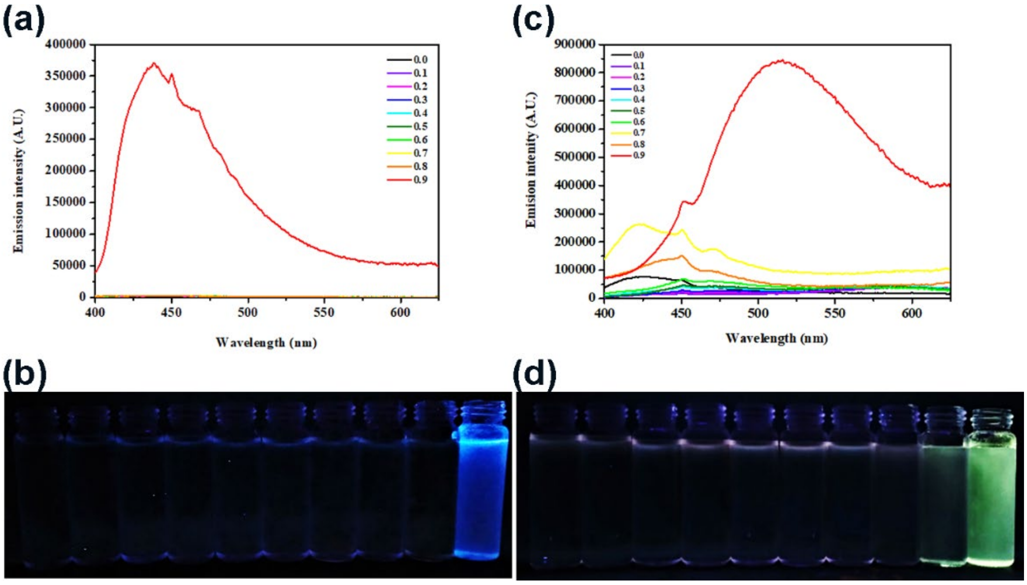

Figure S1. The emission spectra of (a) **CNBrOH** and (b) **CNFCOH** in THF/water mixed solution (5 x 10^{-5} M). The photo of (c) **CNBrOH** and (d) **CNFCOH** in THF/water mixed solution under 365-nm irradiation.

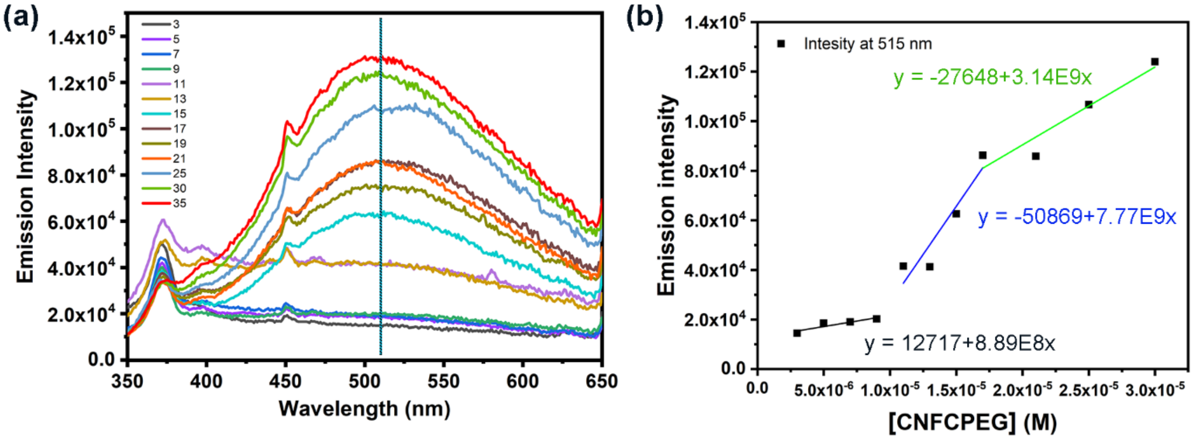

Figure S2. (a) Emission spectra of **CNFCPEG** with various concentration (μM) in the THF/water mixed solution (f_w = 0.8). (b) The emission intensity at 515 nm in different concentration of **CNFCPEG**.

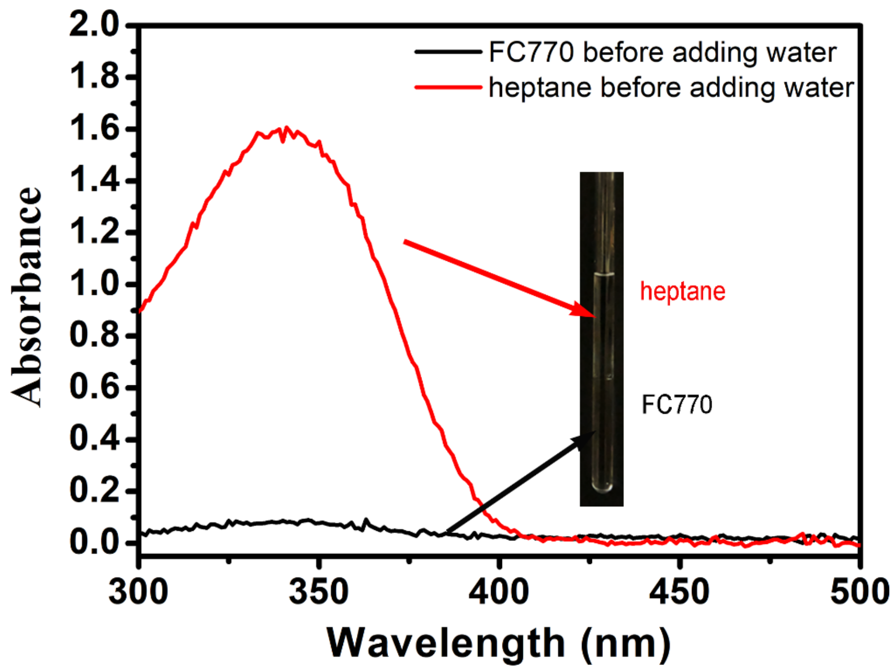

Figure S3. Absorption spectra of **CNFCPEG** (2.5 x 10^{-5} M) in FC770 (black) and heptane (red) before adding water. Inset is the corresponding photo of **CNFCPEG** in the heptane and FC770 mixed solutions.

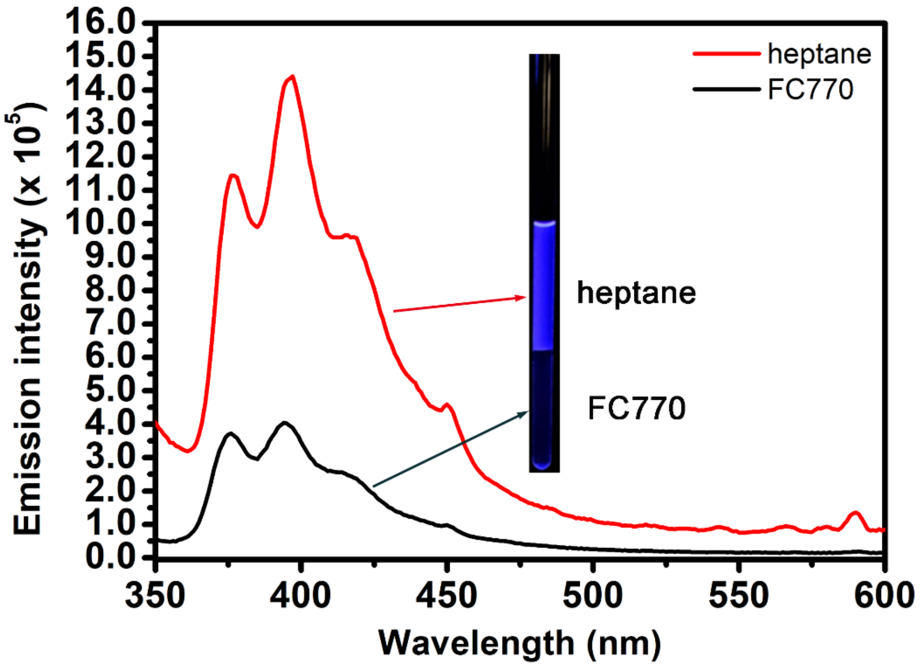

Figure S4. The emission spectra of **CNRFPEG** (2.5 x 10^{-5} M) in FC770 (black) and heptane (red) mixed solutions before adding water.

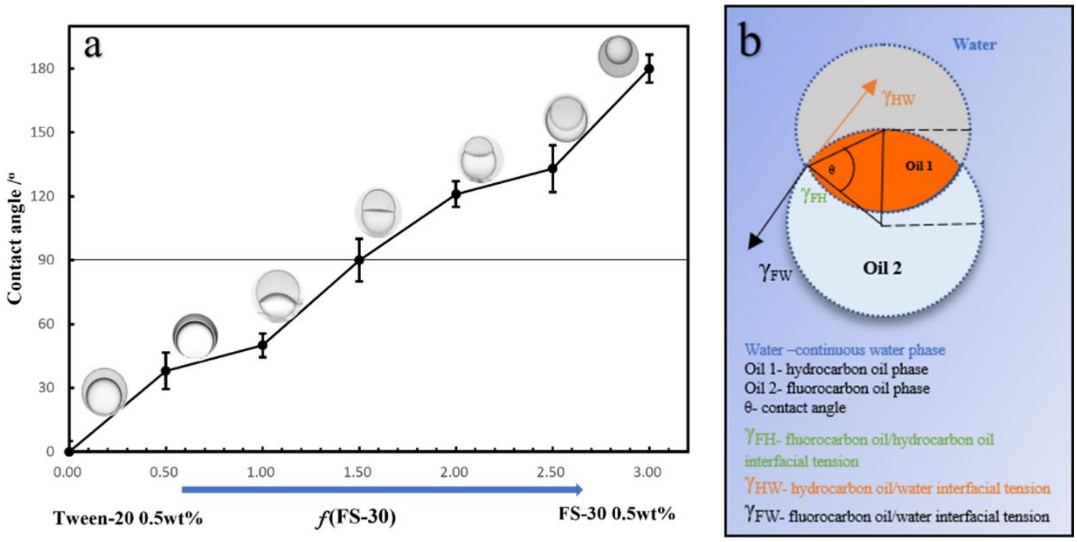

Figure S5. (a) The relation between contact angles and the proportion of FS30 to Tween 20. (b) Schematic representation of the measuring the contact angle technique depends on the interaction area of the two layers of the droplet morphology.

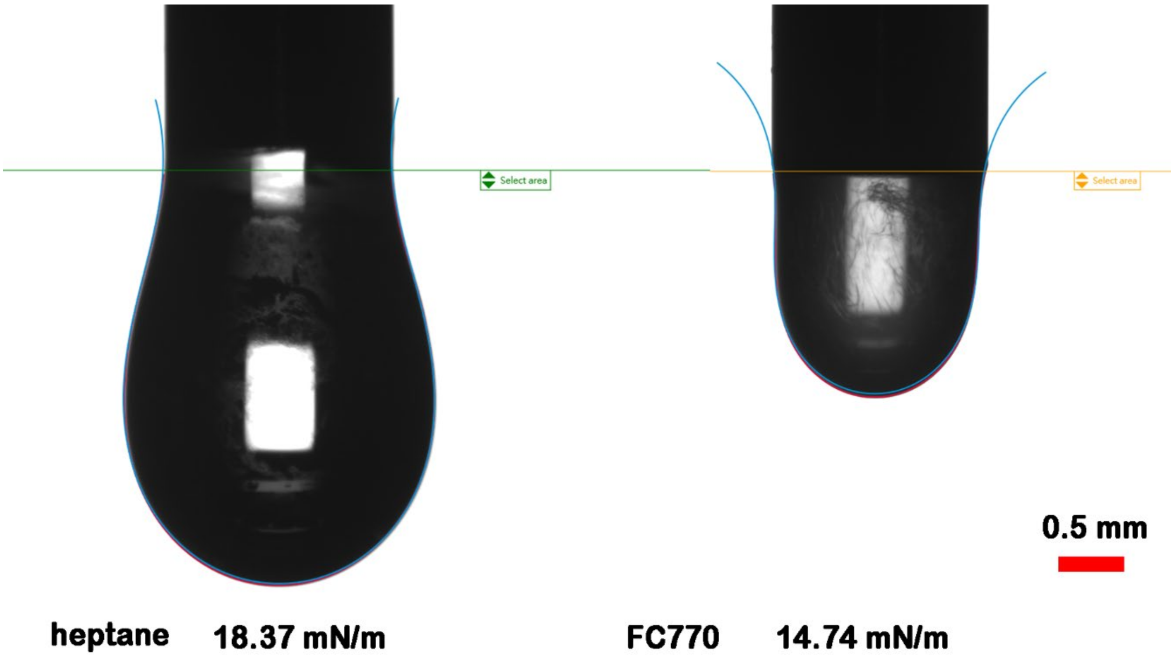

Figure S6. The pendent drop images and surface tension of individual heptane and FC-770 solutions with **CNFCPEG** (5 x 10^{-5} M).

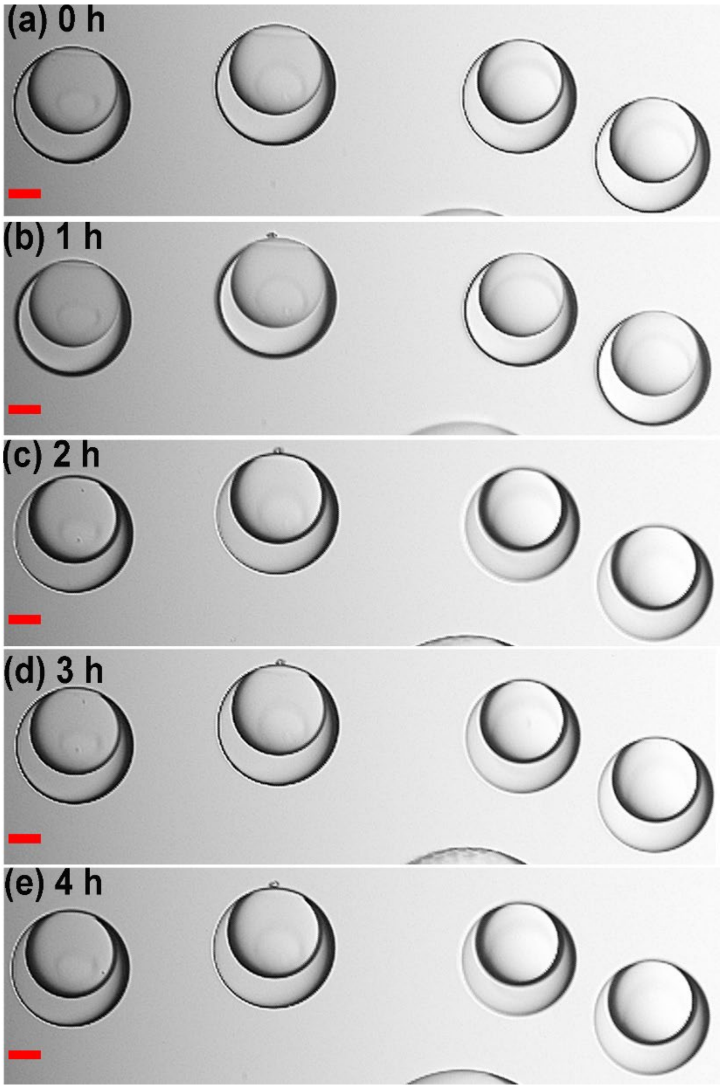

Figure S7. The side-view images of complex emulsions containing **CNFCPEG** (2.5×10^{-5} M) from fresh-prepared to 4 h. (scale bar: 20 μm)

Table S1. The concentration of the iodine$_{(aq)}$, and flow rate, the time of morphology changing from H/F/W to Janus and the estimated iodine content.

entry	[I_2] (mg/mL)	Flow rate (mL/min)	Characteristic time	Iodine (mg)
1	1.0	0.50	1.8 ± 0.3 s	0.015 ± 0.0025
2	0.50	0.50	4.1 ± 0.9 s	0.017 ± 0.0038
3	0.25	0.50	6.2 ± 1.2 s	0.013 ± 0.0025
4	0.10	0.50	8.8 ± 0.8 s	0.0073 ± 0.00067
5	0.05	0.50	16.3 ± 1.6 s	0.0068 ± 0.00067
6	0.025	0.50	25.1 ± 1.5 s	0.0052 ± 0.0003
7	1.0	0.050	1.7 ± 0.03 min	0.085 ± 0.0015
8	0.50	0.050	4.6 ± 0.08 min	0.115 ± 0.002
9	0.25	0.050	6.8 ± 0.06 min	0.085 ± 0.00075
10	0.10	0.050	9.2 ± 0.13 min	0.046 ± 0.00065
11	0.05	0.050	17.6 ± 0.14 min	0.044 ± 0.00035
12	0.025	0.050	27.2 ± 0.18 min	0.034 ± 0.00023

Synthesis details

Synthesis of CNBrOH[S1]

Piperidine (1.7 mL, 18 mmol), and 4-bromophenylacetonitrile (2.35 g, 12 mmol) dissolved in ethanol (20 mL) in a 50-mL round-bottomed flask. The mixture was heated at 78 °C for 30 min. 4-hydroxybenzaldehyde (1.22 g, 10 mmol) dissolved in ethanol (20 mL) was added to the solution, and the mixture was refluxed for 3 hours. After cooling to ambient temperature, the reaction was acidified by adding 18 mL of HCl solution (1 N, 18 mmol). After collecting the solids, the crude products were recrystallized from toluene to afford **CNBrOH** as a yellow solid with a yield of 87%. ^{1}H NMR (400 MHz, DMSO-d_6) δ 7.94 (s, 1H), 7.90–7.84 (m, 2H), 7.72–7.65 (m, 4H), 6.91 (d, J = 6.2 Hz, 2H).

Synthesis of CNFCOH

CNBrOH (0.45 g, 1.5 mmol), sodium acetate (0.47 g, 5.79 mmol), Herrmann's catalyst (0.14 g, 0.15 mmol), and 1H, 1H, 2H-perfluoro-1-decene (0.33 mL, 1.25 mmol) were dissolved in DMF (2.25 mL) in a 10-mL Schlenk tube. The mixture was heated at 125 °C under a nitrogen atmosphere for 24 hours. After cooling to ambient temperature, the mixture was extracted with ethyl acetate and water. The organic layer was washed with brine and dried over anhydrous magnesium sulfate. After removing the solvent under reduced pressure, the crude products were recrystallized from toluene to afford **CNFCOH** as a yellow solid with a yield of 54%. (mp: 161–164 °C) ^{1}H NMR (400 MHz, $CDCl_3$) δ 7.87 (d, J = 8.6 Hz, 2H), 7.69 (d, J = 8.3 Hz, 2H), 7.61–7.48 (m, 3H), 7.19 (d, J = 16.4 Hz, 1H), 6.94 (d, J = 8.6 Hz, 2H), 6.31–6.19 (m, 1H), 5.49 (s, 1H). ^{13}C NMR (101 MHz, DMSO-*d6*) δ 160.3, 143.1, 138.9, 138.8, 135.9, 132.9, 131.6, 129.2, 128.3, 125.4, 124.5, 118.1, 118.0, 115.9, 115.1(m), 114.0(m), 112.8(m), 110.3(m), 107.7(m), 104.9. ^{19}F NMR (377 MHz, DMSO-d_6) δ -80.13 (t, J = 7.5 Hz, 3F), -109.46 (m, 2F), -121.05(m, 2F), -121.57(m, 4F), -122.42 (m, 4F), -125.61(m, 2F). HRMS (ESI) calcd for $C_{25}H_{13}F_{17}NO^+$ ($M+H^+$):666.0726 found:666.0749.

Synthesis of tetraethylene glycol mono methyl ether tosylate (m-PEG5-Tos)[S2]

Tetraethyleneglycol monomethyl ether (2.10 mL, 10 mmol), triethylamine (0.16 mL, 15 mmol), 4-dimethylaminopyridine (0.122 g, 1 mmol) were dissolved in 10 mL DCM in a 50-mL Schlenk flaks. To the mixture, tosyl chloride (2.859 g, 15 mmol) dissolved in 10 mL DCM was slowly added under a nitrogen atmosphere at 0 °C. The mixture was stirred at ambient temperature for 24 hours. After removing the solvents under reduced pressure, the residue was purified by column chromatography (DCM:MeOH = 100:4) to afford the m-PEG5-Tos as light-yellow oil with a yield of 33%. ^{1}H NMR (400 MHz, Chloroform-d) δ 7.80 (d, 2H), 7.34 (d, J = 8.1 Hz,

2H), 4.16 (t, 2H), 3.70 – 3.67 (m, 2H), 3.63 (s, 6H), 3.58 (s, 4H), 3.56 – 3.52 (m, 2H), 3.37 (s, 3H), 2.45 (s, 3H).

Synthesis of CNFCPEG

CNFCOH (0.63 g, 0.94 mmol), m-PEG5-Tos (0.16 g, 0.79 mmol), and potassium carbonate (0.32 g, 2.37 mmol) were dissolved in 7 mL anhydrous DMF in a 25-mL Schlenk flask. The mixture was heated at 45 °C under a nitrogen atmosphere for 24 h. The mixure was extracted by ethyl acetate and water. The organic layer was washed with 1 N NaOH aqueous solution and brine. After removing the solvents under reduced pressure, **CNFCPEG** was obatined as a yellow solid with a yield of 33%. (mp = 41–42 °C) ^{1}H NMR (400 MHz, Chloroform-d) δ 7.90 (d, *J* = 9.0 Hz, 2H), 7.69 (d, *J* = 8.4 Hz, 2H), 7.58–7.48 (m, 3H), 7.19 (d, *J* = 15.7 Hz, 1H), 7.00 (d, *J* = 8.9 Hz, 2H), 6.32–6.18 (m, 1H), 4.24–4.17 (m, 2H), 3.93–3.86 (m, 2H), 3.78–3.71 (m, 3H), 3.67 (m, 9H), 3.58–3.51 (m, 2H), 3.38 (s, 3H). ^{13}C NMR (100 MHz, $CDCl_3$) δ 161.1, 142.6, 138.84, 138.75, 136.6, 133.8, 131.5, 128.3, 126.4, 126.2, 125.8, 118.3, 115.1, 115.0, 114.9, 107.6, 72.0, 70.9, 70.68, 70.66, 70.6, 69.6, 67.7, 59.1. ^{19}F NMR (377 MHz, DMSO-d6) δ -80.15(t, 3F), -109.52 (m, 2F), -121.06(m, 2F), -121.58(m, 4F), -122.20(m, 4F), -122.67 (m, 4F), -125.64 (m, 2F). HRMS (ESI) calcd for $C_{34}H_{31}F_{17}NO_5^+$ (M+H$^+$):856.1931 found:856.1930.

[S1] M. Kondo, T. Yamoto, S. Miura, M. Hashimoto, C. Kitamura, N. Kawatsuki, *Chem. - Asian J.* **2019**, *14*, 471.

[S2] M. R. J. Vallée, P. Majkut, I. Wilkening, C. Weise, G. Müller, C. P. R. Hackenberger, *Org. Lett.* **2011**, *13*, 5440.

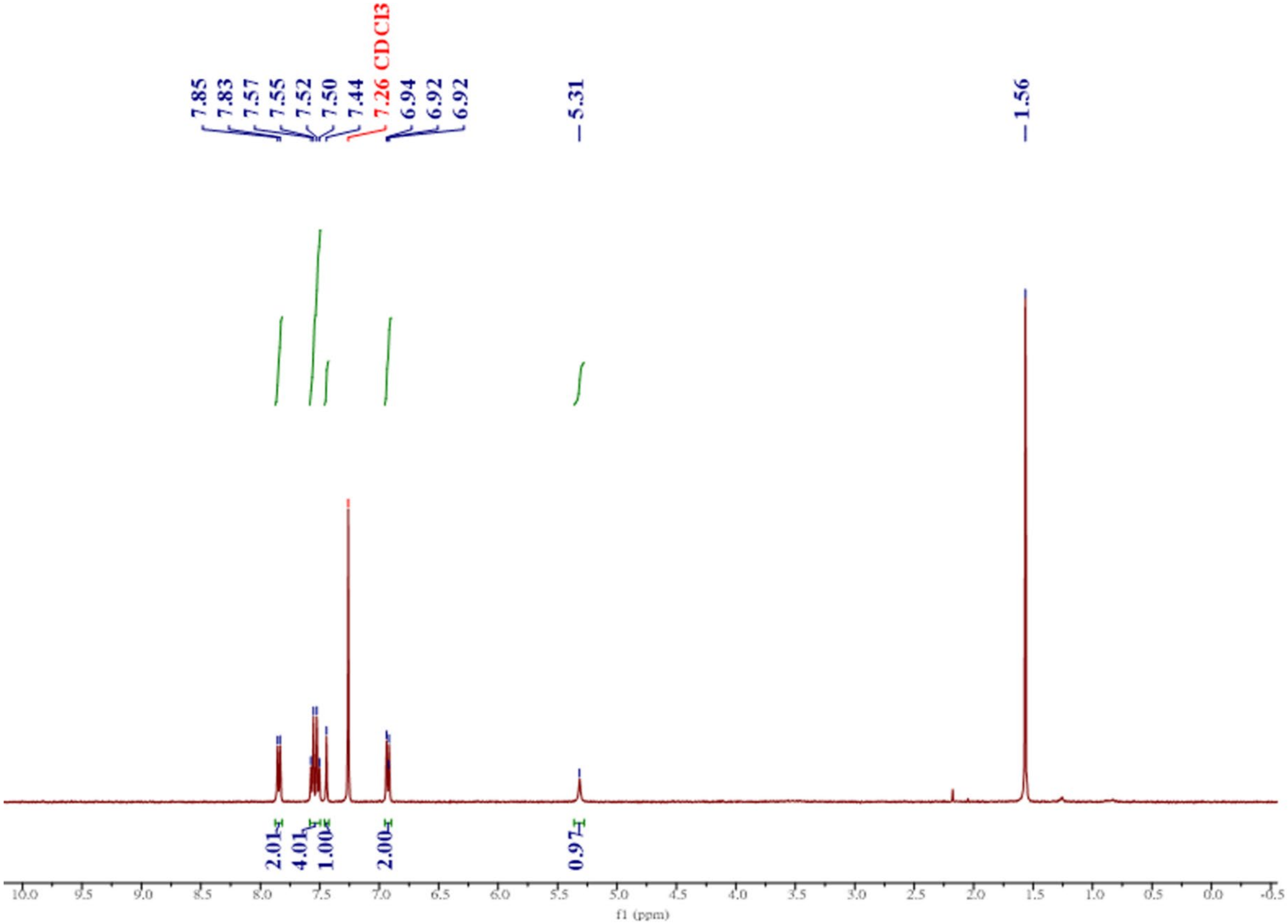

Figure S8. ^{1}H NMR spectrum of **CNBrOH** in $CDCl_3$

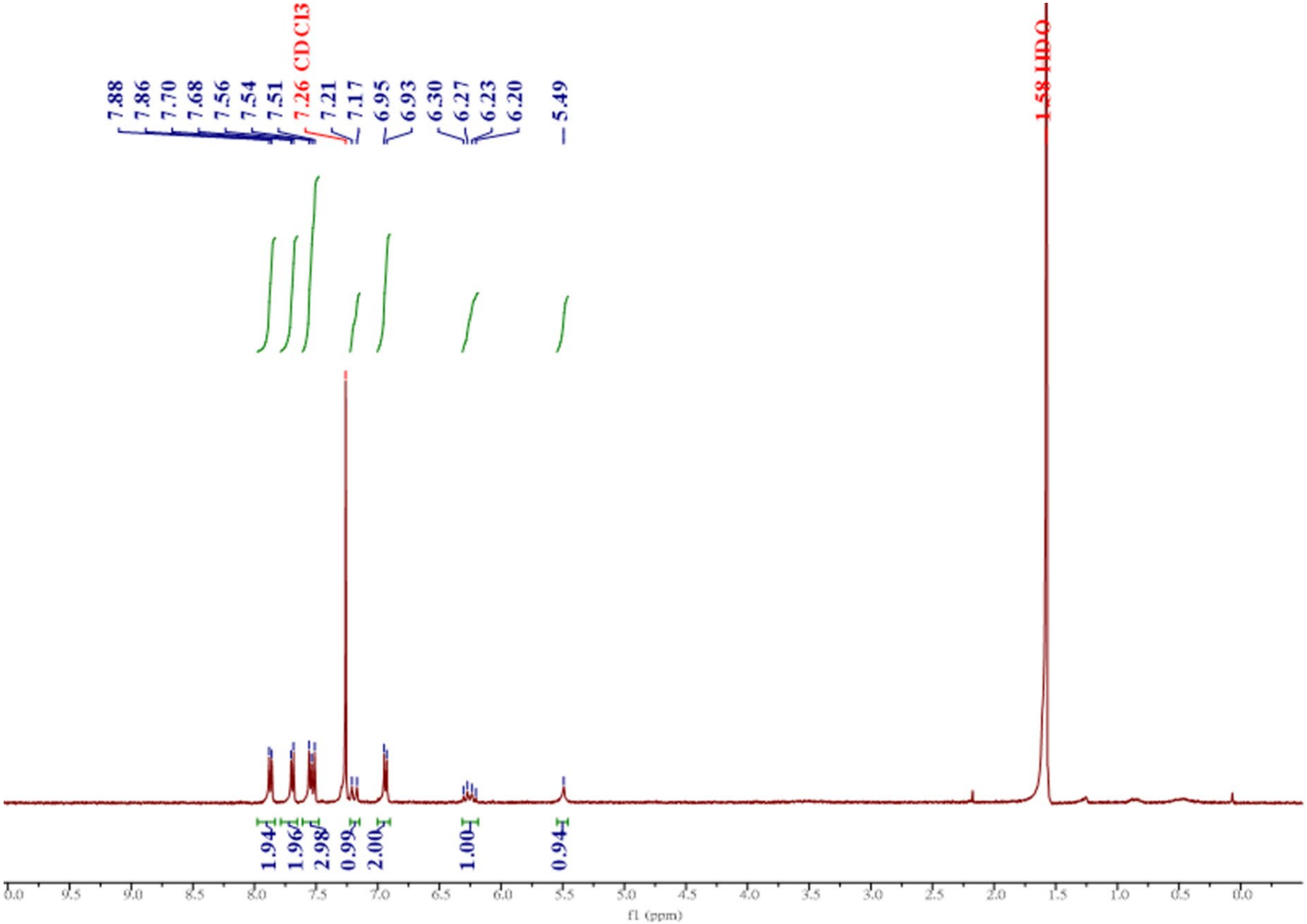

Figure S9. ^{1}H NMR spectrum of **CNFCOH** in $CDCl_3$

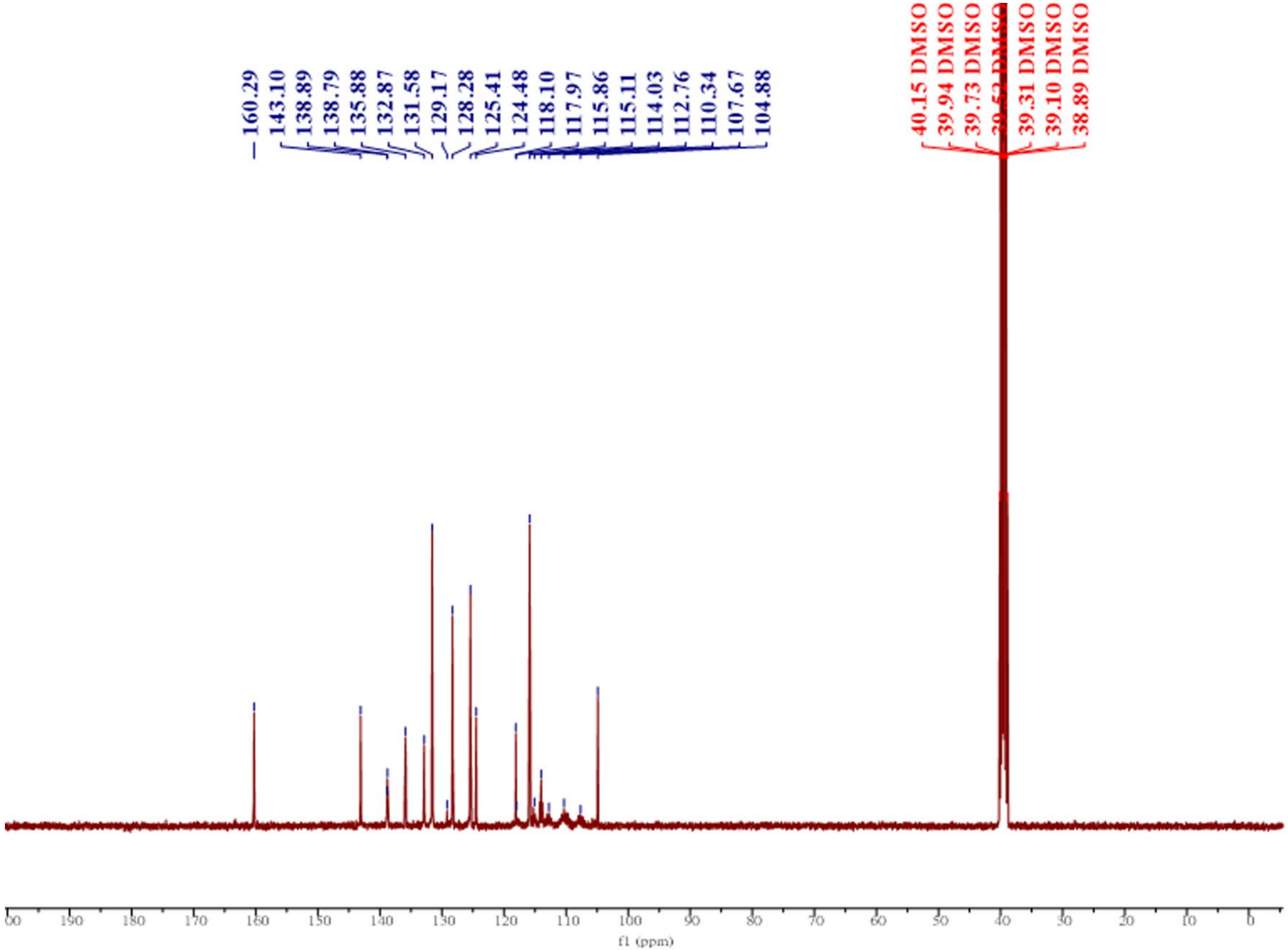

Figure S10. ^{13}C NMR spectrum of **CNFCOH** in DMSO-*d6*

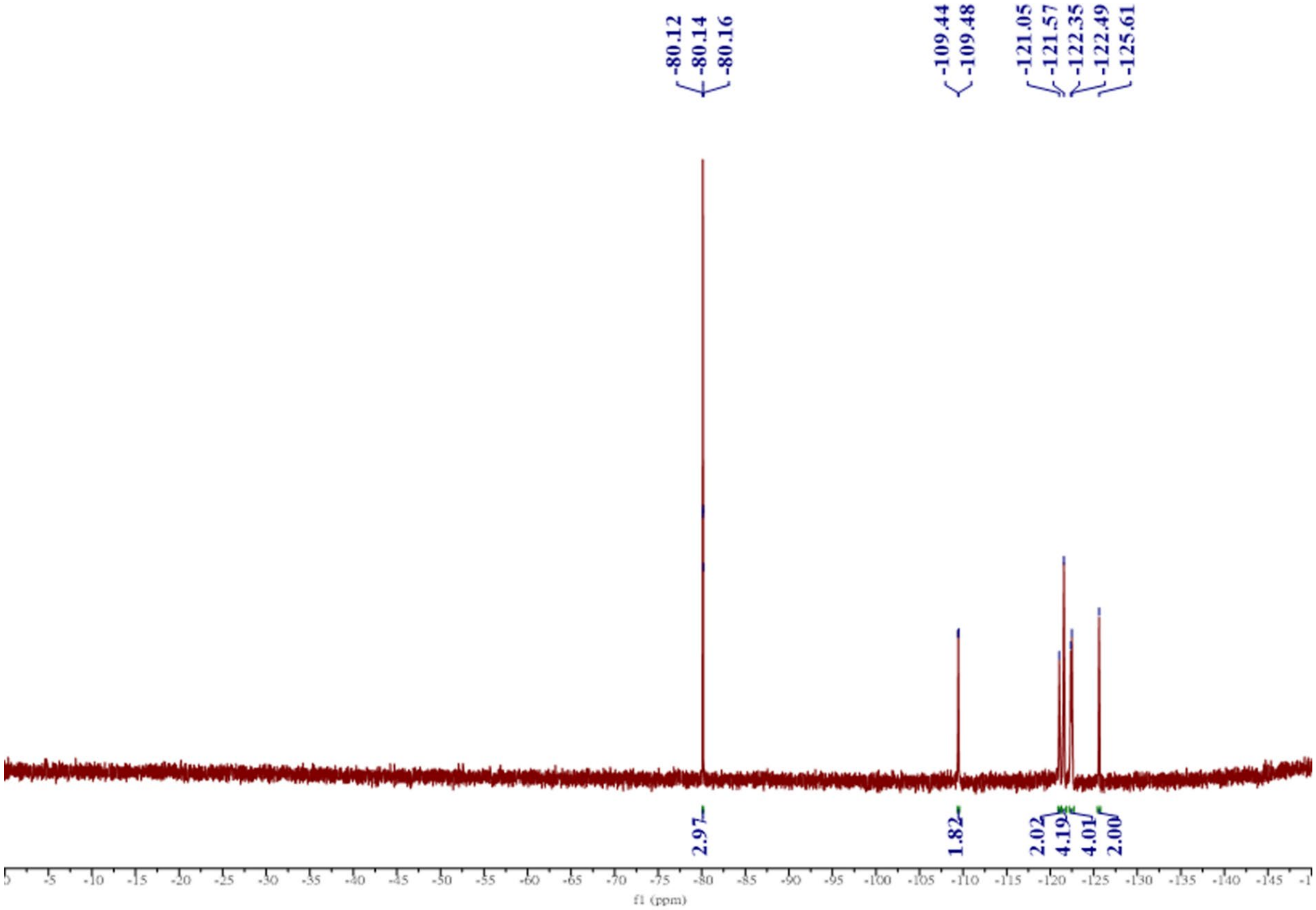

Figure S11. ^{19}F NMR spectrum of **CNRFOH** in DMSO-*d*6

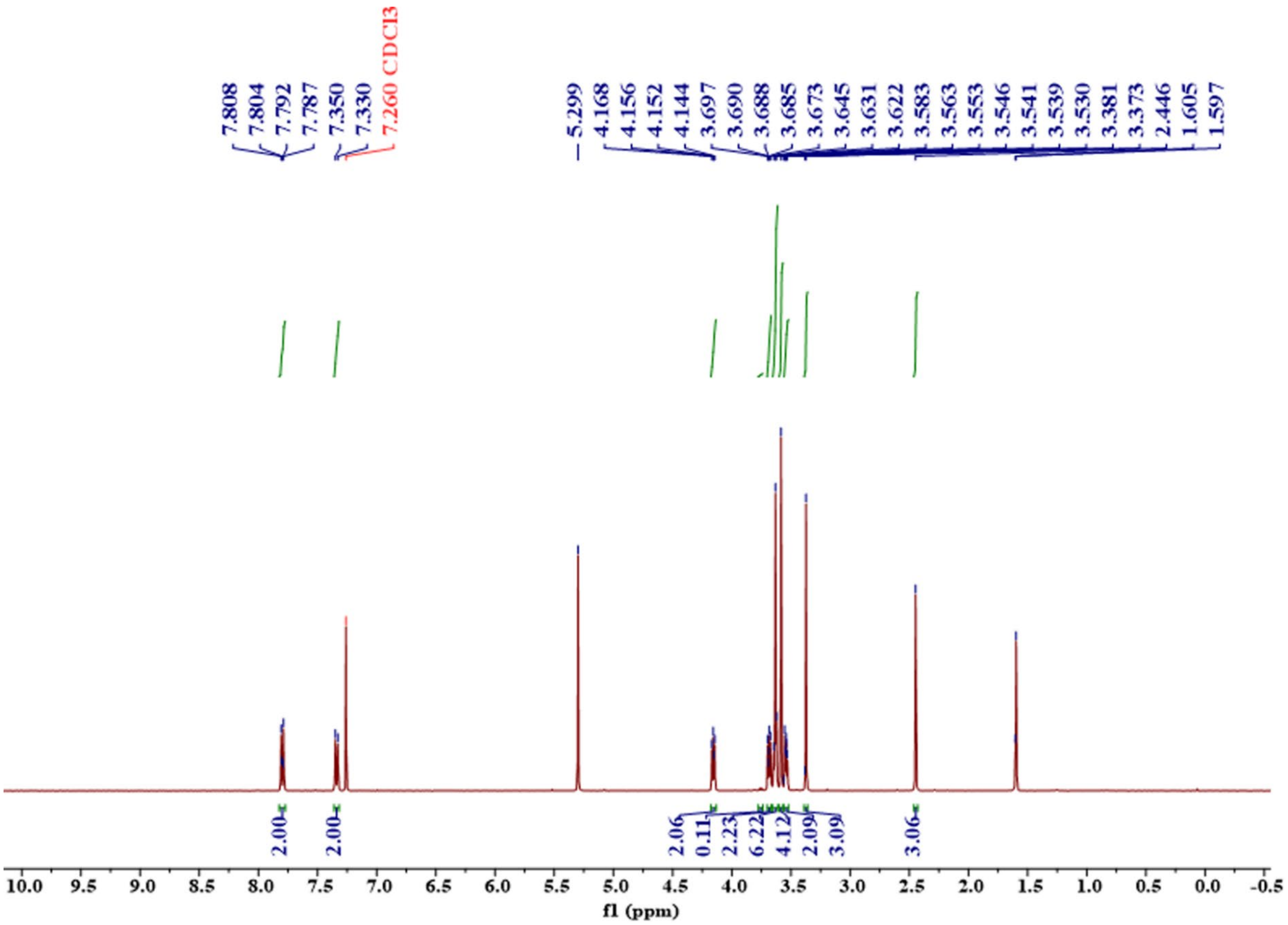

Figure S12. ^{1}H NMR spectrum of m-PEG5-Tos in $CDCl_3$

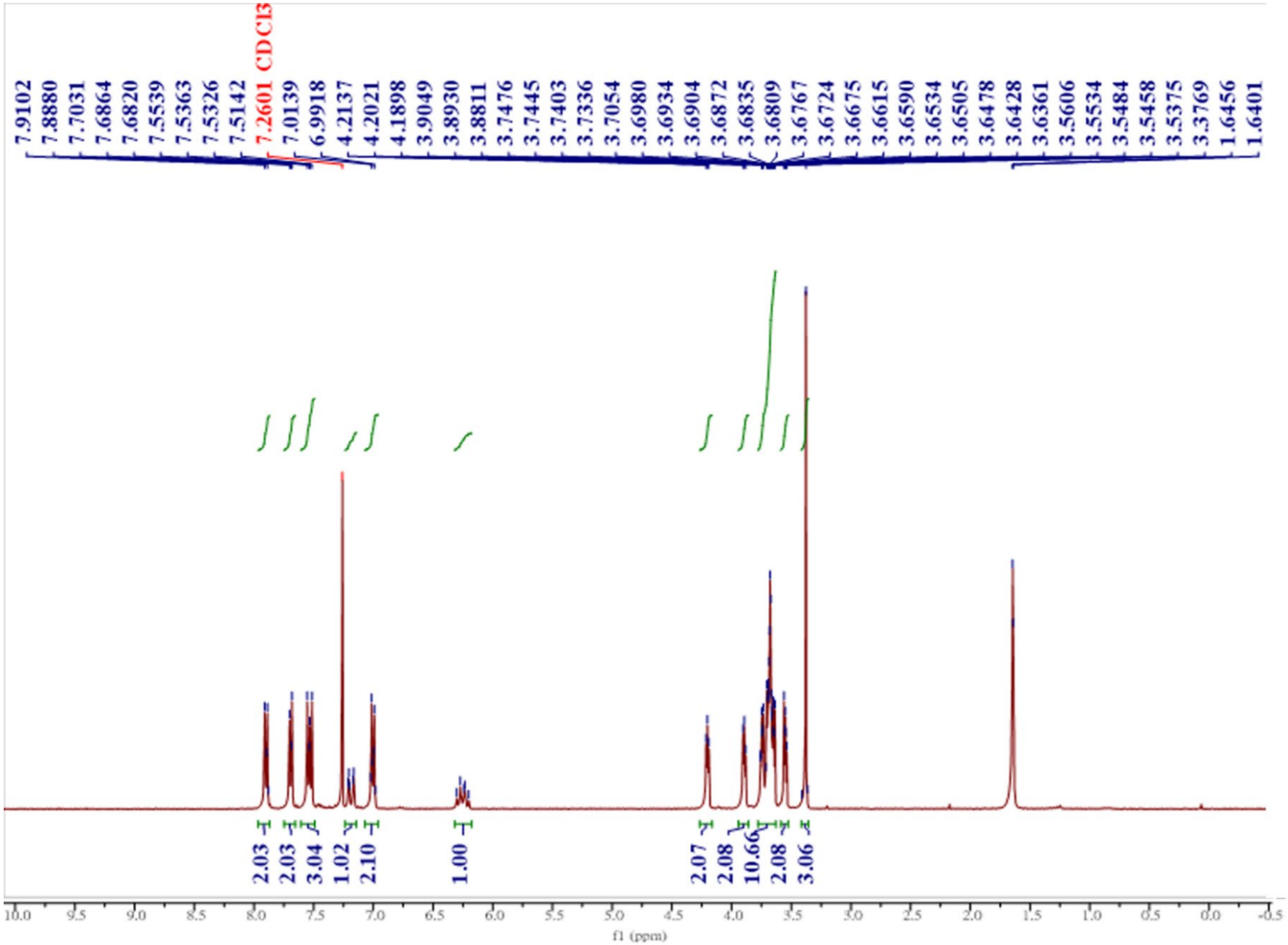

Figure S13. ^{1}H NMR spectrum of **CNFCPEG** in $CDCl_3$

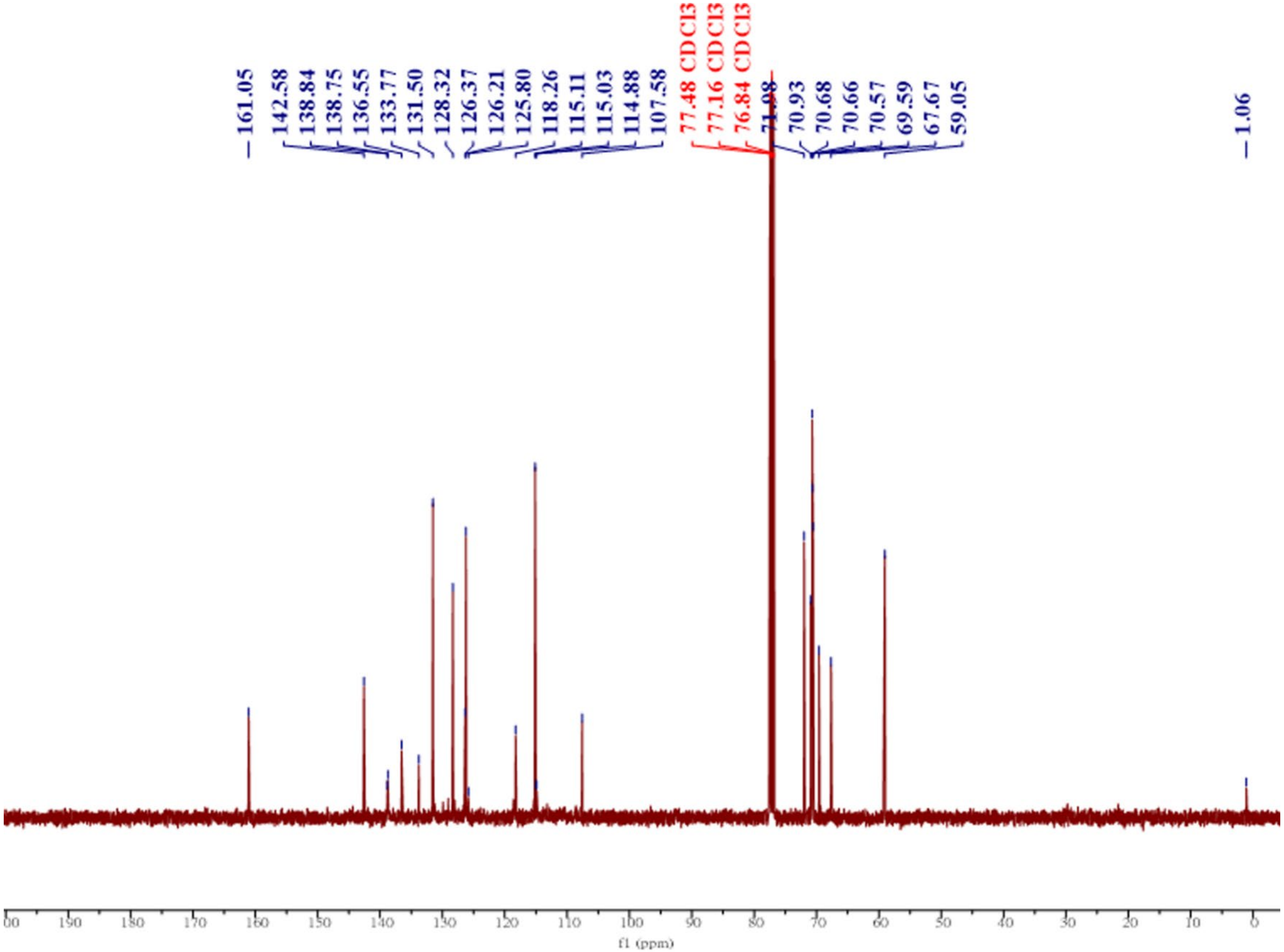

Figure S14. ^{13}C NMR spectrum of **CNFCPEG** in $CDCl_3$

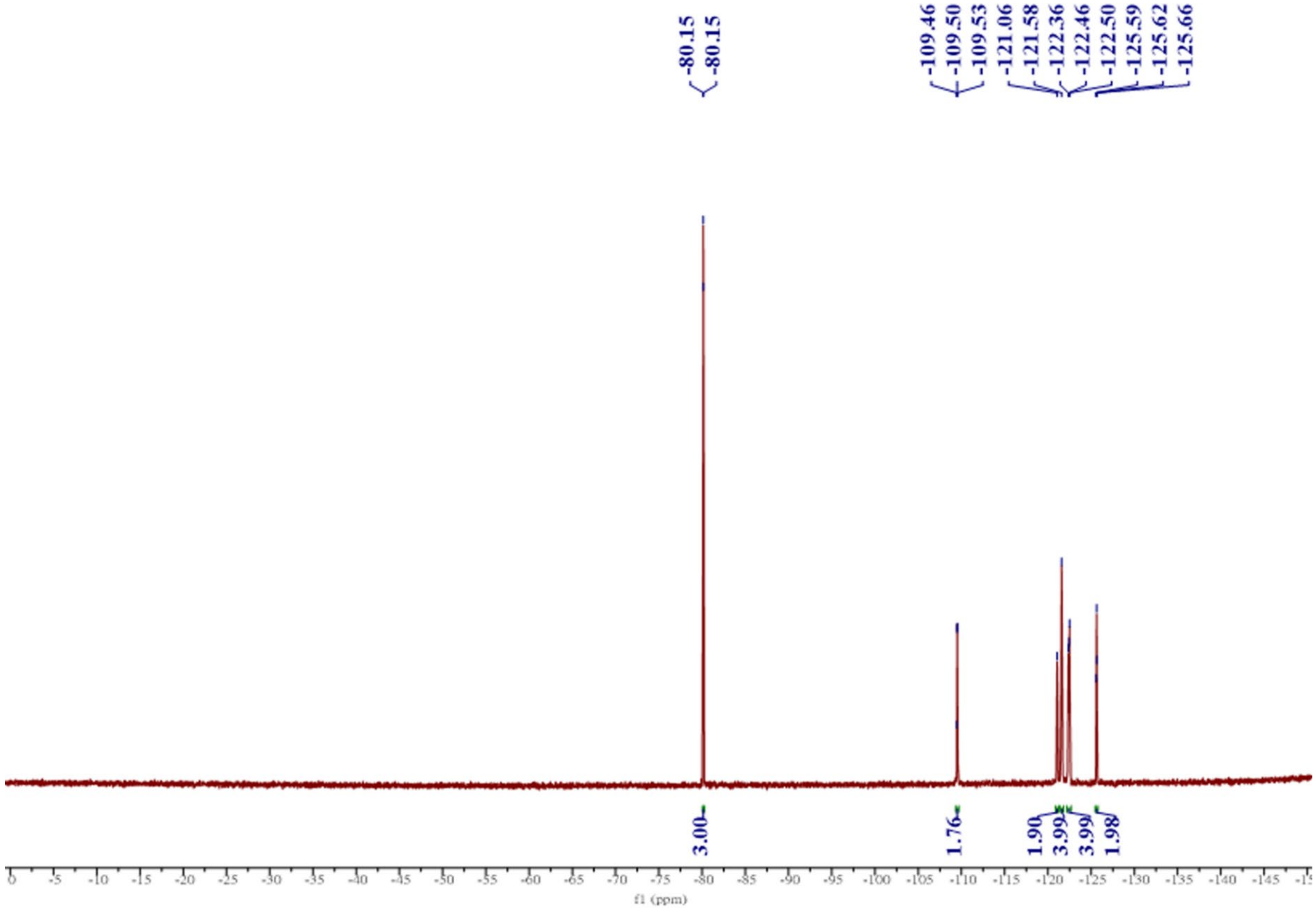

Figure S15. ^{19}F NMR spectrum of **CNRFPEG** in DMSO-*d*6
